# Hypermanganesemia Induced Chorea and Cognitive Decline in a Tea Seller

**DOI:** 10.5334/tohm.537

**Published:** 2020-10-20

**Authors:** Ritwik Ghosh, Souvik Dubey, Subhankar Chatterjee, Mrinalkanti Ghosh, Biman Kanti Ray, Julián Benito-León

**Affiliations:** 1 Department of General Medicine, Burdwan Medical College and Hospital, Burdwan, West Bengal, IN; 2 Department of Neuromedicine, Bangur Institute of Neurosciences, Kolkata, IN; 3 Department of General Medicine, Rajendra Institute of Medical Sciences, Ranchi, IN; 4 Department of Radiology, Burdwan Medical College, Burdwan, West Bengal, IN; 5 Department of Neurology, University Hospital “12 de Octubre”, Madrid, ES; 6 Centro de Investigación Biomédica en Red sobre Enfermedades Neurodegenerativas (CIBERNED), Madrid, ES; 7 Department of Medicine, Complutense University, Madrid, ES

**Keywords:** Hypermanganesemia, Manganese, Chorea, Movement disorder, Dementia, Cognition, Tea

## Abstract

**Background:**

Manganese associated neurotoxicity and neurodegeneration is quite rare yet established neurological disorder. This neurotoxic element has predilection for depositing in basal ganglia structures, manifesting mainly as parkinsonian and dystonic movement disorders with behavioral abnormalities.

**Case report:**

We report a 40-year-old man who presented with a subacute onset bilateral, asymmetric hyperkinetic movement disorder (predominantly left sided chorea) with multi-domain cognitive impairment, dysarthria, and generalized rigidity. Clinical history and examination yielded multiple differential diagnoses including deposition and metabolic disorders, autoimmune and paraneoplastic encephalitis involving basal ganglia, and neurodegenerative disorders with chorea and cognitive impairment. However, magnetic resonance imaging was suggestive of paramagnetic substance deposition, which came out to be manganese after laboratory investigations. History, clinical examinations, and investigation results pointed towards a diagnosis of acquired hypermanganesemia due to over-ingestion of manganese containing substance (i.e., black tea). He was treated symptomatically and with chelation therapy (calcium disodium edetate). At the sixth month of follow-up, complete resolution of chorea, dysarthria and partial amelioration of rigidity were observed. His cognitive decline and behavioral abnormalities improved.

**Discussion:**

This is probably the first reported case of acquired hypermanganesemia that presented as a combination of asymmetric chorea and cognitive dysfunction with atypical imaging characteristics. The clinical picture mimicked that of Huntington’s disease. We highlight the potential deleterious effects of an apparently “benign” non-alcoholic beverage (i.e., black tea) on cerebral metabolism.

## Introduction

Manganese is an essential trace element having crucial role in cellular metabolism, but, when in excess, can lead to serious neurotoxicity, which can be accredited to mitochondrial dysfunction, injury to dopaminergic, serotonergic, GABAergic and glutaminergic neurotransmission, free radical damage, and neuroinflammation [1]. Manganese affects neurons of striatum (caudate nucleus, putamen and nucleus accumbens), globus pallidus and substantia nigra, resulting in motor dysfunction with associated psychiatric and cognitive features, collectively known as “manganism” [2,3,4]. Hypermanganesemia due to mutations in SLC30A10, SLC39A14, and SLC39A8 presents at an early age with predominantly dystonia, gait disturbance, blood dyscrasias, and hepatic dysfunction [5,6,7,8,9,10,11]. On the other side, common causes of acquired hypermanganesemia are dietary exposure (food and drinking water), inhalational exposure, total parenteral nutrition and recreational drug use, among others [12,13,14,15]. However, magnetic resonance imaging (MRI) features of both types of manganese deposition are similar (i.e. increased signal intensity on T1-weighted images in basal ganglia) [7,11].

We herein report a middle-aged man who presented with subacute onset cognitive decline and chorea caused by hypermanganesemia probably due to chronic compulsive over consumption of manganese containing drink (i.e., black tea).

## Case presentation

A 40-year-old right-handed man, who worked as a tea seller and had 12 years of formal education, was admitted to the hospital reporting gradual onset progressive dance-like movements for last 2 months and deterioration of mental status for last 1 month. The patient was unable to provide an accurate timeline of events leading up to his presentation. According to his wife, the patient had conversation difficulties and misplacement of objects associated with recent memory impairment and behavioral abnormalities (aggression, compulsive behavior and disinhibition).

His past medical history and drug history were unrevealing. He had no history of smoking, drug addiction and reported only occasional alcohol use, but had craving for tea. For last 10–15 years he used to drink on an average of 20 cups of black tea [150 ml (5 Oz) of tea per cup] per day. His wife noticed a significant increase in this craving for tea over last few months. Craving for tea became so severe that his family members had to shut down his tea-stall eventually.

On examination, he had severe dysarthria and impaired verbal fluency with apparently unaffected comprehension. Mini Mental Status Examination (MMSE) total score was 16/30. Complex attention, executive function, social cognition, learning, and memory domains were affected. He had normal power in all four limbs with generalized rigidity and asymmetric chorea (predominantly involving left upper and lower limbs) (video). Deep tendon reflexes were normal with bilateral flexor plantar responses. He had walking difficulties due to persistent abnormal movements. Saccades were poorly initiated and hypometric with fractionated smooth pursuit. All other aspects of neurological examination and review of other systems were normal.

**Video:** **Asymmetric choreic movements in a case of acquired hypermanganesemia.** Video demonstrating involuntary, rapid dance-like movements involving predominantly left upper and lower limbs that flow from one muscle to the other in continuous fashion suggestive of a predominantly left hemichorea (Although the patient had bilateral chorea, left much more than right with motor impersistence, this is not demonstrated in the video).

**Figure 1:**
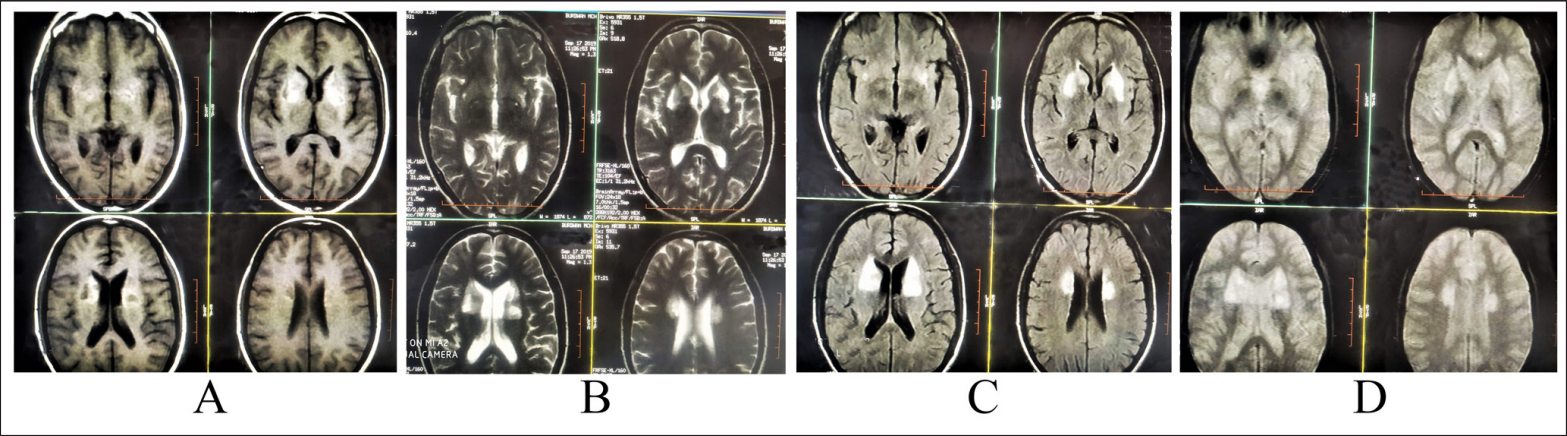
Brain MRI shows non-enhancing bilateral increased signal intensity on axial T1-weighted **(A)**, axial T2-weighted **(B)** and axial T2-fluid-attenuated inversion recovery (FLAIR)-weighted **(C)** images in basal ganglia, with scattered areas of gliosis (T1-weighted images). Gradient-echo axial T2*-weighted image **(D)** shows no evidence of signal blooming (unlike iron or calcium deposition, manganese deposition does not cause blooming on gradient-echo images or susceptibility-weighted imaging).

Complete blood count, along with peripheral blood smear, renal, liver, thyroid function tests, blood glucose, serum sodium, potassium, calcium, phosphorus, lactate, and iron profile were within normal limits. Brain MRI revealed non-enhancing, bilateral symmetrical increased signal intensity on T1-weighted, T2-weighted and T2-weighted-fluid-attenuated inversion recovery (FLAIR) images in basal ganglia (particularly involving caudate nucleus and putamen), with areas of gliosis, without any mass effect (Figure 1). Electroencephalogram was normal. Deposition and metabolic disorders, autoimmune and paraneoplastic encephalitis, and Huntington’s disease were considered as differential diagnoses. HIV serology, autoimmune and paraneoplastic neuronal antibodies panel [NMDA (anti-glutamate receptor against NR1 subunit), AMPA (anti-glutamate)-GluR1, AMPA (anti-glutamate)-GluR2, GABA-B receptor antibody, LGI-1 antibody, CASPR2 antibody, amphiphysin, anti-CRMP5/CV2, PNMA2 (Ma2/Ta), ANNA-1/Hu, ANNA-2/Ri, and PCA-1/Yo antibodies], amino acid chromatography and routine cerebrospinal fluid (CSF) (cell type, cell count, protein, glucose, bacterial staining, and cultures), assay for protein 14-3-3 in CSF, and genetics for Huntington’s disease and Huntington’s disease like (HDL) 4/SCA 17 were all normal or negative. Serum ceruloplasmin, copper, 24 hours urinary copper, and test for ATP7B gene were normal. Slit lamp examination ruled out Kayser-Fleischer ring. MR portography was normal.

The serum manganese level was sent, which came out to be 3281 nmol/L (reference value < 320 nmol/L). Although inherited manganism was unlikely, considering the age of the patient and negative family history, screening for mutations for SLC39A14, SLC30A10 and SLC39A8 were done for objective confirmation and expectedly came out to be negative. Personal history revealed he had a compulsive habit of drinking black tea, on an average 20 cups (5 Oz/cup) per day over last 10–15 years. On an average he ingested 26 mg of manganese per day for last 10–15 years.

Edetate calcium disodium was administered as a chelation therapy at 1 g/m^2^/day (in 500 ml normal saline) once daily for 5 days. Further, oral tetrabenazine (50 mg/day) was given. On tenth day, the serum manganese level decreased (1070 nmol/L) and motor symptoms started showing some improvement. Intravenous chelation with edetate calcium disodium was repeated twice in first month (two weeks apart) and once in third month. Metabolic parameters were routinely monitored alongside serum manganese level (Figure 3). After three cycles of chelation therapy, his serum manganese level normalized (189 nmol/L) and we then stopped chelation therapy. Tetrabenazine was also stopped at four weeks of follow-up as there was no abnormal movement.

**Figure 2:**
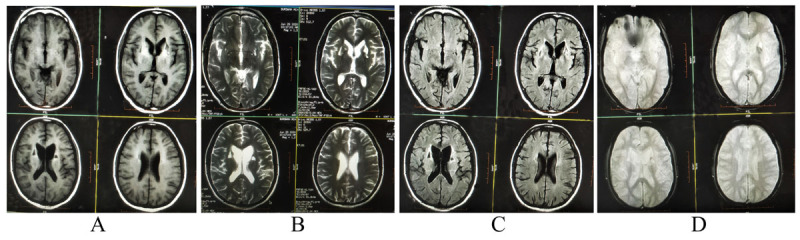
Follow-up brain MRI after 6 months: Axial T1-weighted image **(A)** shows heterogenous signal changes (i.e. bilateral increased signal intensity in basal ganglia possibly due to manganese deposition along with hypointense gliotic areas with mild dilatation of frontal horns of both lateral ventricles). Axial T2-weighted **(B)** and T2-FLAIR-weighted **(C)** images show iso- to hypointense signal changes at the areas of deposits and scattered gliotic foci with central hypo and peripheral hyperintense signal changes. Gradient-echo axial T2*-weighted image (D) shows no evidence of signal blooming.

**Figure 3:**
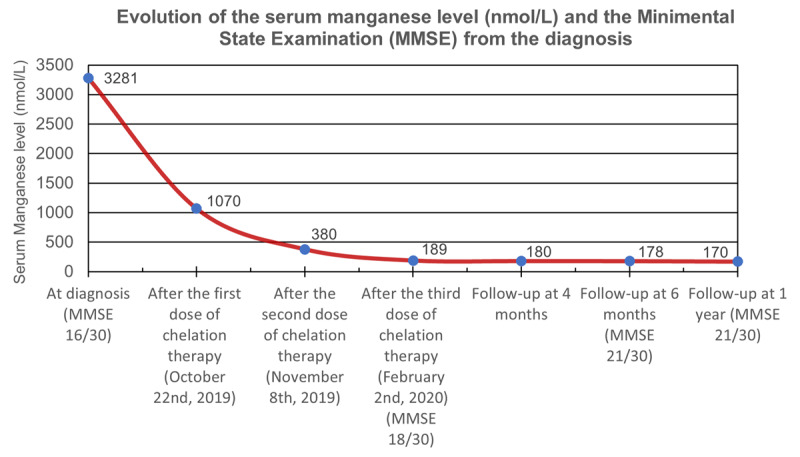
Schematic diagram of the timeline of events in relation to serum manganese level and Mini Mental Status Examination (MMSE) along time.

At the sixth month of follow-up, complete resolution of chorea, dysarthria and partial amelioration of rigidity were observed. Serum manganese level remained normal (178 nmol/L). Cognitive function improved in the following domains: complex attention, executive function, social cognition, learning and memory. MMSE score improved by 5 points (i.e. 21/30). Brain MRI, at follow-up, revealed partial resolution of previous findings, but the gliotic areas, which persisted along with resultant dilatation of frontal horns of lateral ventricles due to negative mass effects (Figure 2). At the one year of follow-up, the patient’s clinical status remained similar and had a normal serum manganese level (170 nmol/L). Figure 3 summarizes the timeline of events in relation to serum manganese level and MMSE along time.

## Discussion

Disruption anywhere in the circuitries between frontal cortex and structures of basal ganglia may lead to neurocognitive and behavioral dysfunction along with movement disorders [16,17] as evident in the current case. Thus, involvement of basal ganglia and its cortical connections should be suspected in patients with subacute onset chorea followed by multi-domain cognitive decline [16,17]. Considering age, form of presentation and neuroimaging characteristics, exclusion of deposition disorders was mandatory. With this scenario, calcium, copper, iron, and manganese deposition disorders were considered. Serum calcium was normal and brain CT scan did not reveal any calcium deposition. Tremor, dystonia, and parkinsonism are far more common than pure chorea in Wilson disease [18]. A complex and heterogenous spectrum of inherited neurodegenerative disorders, the neurodegeneration with brain iron accumulation (NBIA) disorders, are characterized by superabundant iron accumulation, particularly in the basal ganglia, movement disorders (usually dystonia and parkinsonism), retinal abnormalities and cognitive impairment [19]. Extensive clinical and laboratory investigations ruled out both Wilson disease and NBIA propelling us to think of some other deposition disorder than copper, iron and calcium.

Subacute onset chorea followed by cognitive impairment warranted investigations for Huntington’s disease and HDL disorders. Family history and genetics were negative for Huntington’s disease and HDL 4/SCA 17. Relative rarity of HDL in eastern India (permanent residence of the patient), absence of positive family history, age of presentation, and significant response to chelation therapy (in terms of clinical, biochemical and neuroradiological improvements) restrained us from doing costly genetic testing for HDL1,2 and 3. Clinical-radiological manifestations were better explained by an alternative etiology, supported by other investigations (i.e. high serum manganese level).

A neuroacanthocytosis syndrome was a very unlikely possibility by absence of peripheral neuropathy, raised creatinine kinase and acanthocytes in peripheral blood smears. Mitochondrial cytopathies were also an exceedingly rare possibility by normal serum and cerebrospinal fluid lactate and normal lactate-pyruvate ratios. In addition, the clinical picture improved after chelation therapy.

After ruling out possible and relevant differential diagnoses and finding extremely high serum manganese level, deposition of manganese in both basal ganglia was presumed to be the explanation of the clinical picture. Typical movement disorders in manganese deposition are dystonia, dystonic tremor, and parkinsonism [9]. Chorea or choreoathetosis, although uncommon, have been reported [20]. Fell et al. [21], described two children receiving long-term parenteral nutrition with manganese toxicity who developed dystonic limb movements and abnormal posturing. Haug et al. [22], reported an interesting case of Morvan’s fibrillary chorea in which chronic manganese intoxication (occupational exposure) seemed to be implicated. Manganese inhibits acetylcholine esterase and thus could lead to generation of central and peripheral cholinergic activity [22]. In a survey carried out in a dry battery industry with high inhalational exposure to manganese revealed that 2 out of 36 workers had movement disorders (one with left hemi-parkinsonism and other having left hemi-choreoathetosis) [23]. Although among basal ganglia nuclei, globus pallidus is affected preferentially, other areas such as striatum, subthalamic nuclei, substantia nigra and thalami can be affected in majority of the cases of manganism [24,25]. Classically, hypermanganesemia is associated with bilateral increased signal intensity on T1-weighted images in the basal ganglia and iso- to hyperintense signal changes on T2-weighted images [11,26]. Increased signal intensity on T2-weighted in basal ganglia, similar to the present case, although rare, has been reported previosly [26]. A probable explanation of this fact is astrogliosis and spongiform neurodegeneration due to prolonged manganese neurotoxicity [27]. Moreover, we excluded many other possible causes of bilateral increased signal intensity on T2-weighted images in the basal ganglia basing on clinical grounds and appropriate investigations.

Searching for probable etiologies of manganese deposition, hepatic causes were excluded (normal liver function tests and normal MR portography). Inhalational exposure was not evident. Common genetic causes of hypermanganesemia were also ruled out. Hence, hypermanganesemia of our patient could be due to consumption of manganese containing food or unknown exposure to some toxins containing manganese. History was revisited, which revealed patient had habit of compulsive black tea intake. The serum manganese level in our patient was found to be similarly high compared to other recently reported cases of hypermanganesemia, i.e. above 3000 nmol/L, which was far beyond the reference value (less than 320 nmol/L) [4,5,7,10].

Manganese, an essential trace element, is neurotoxic at high doses [1,2,3,4,28]. Excess dietary intake with food and/or drinking water, and inhalational exposure from working places are considered common sources of environmental exposure [12,13,14,15,28]. Chronic exposure to excess manganese from drinking water (≥0.2 mg/L) has been linked to poor scholastic performance, impaired cognitive abilities, neurobehavioral dysfunction, oppositional behavior, hyperactivity and manganism in children [28]. Indian black tea is rich in manganese (range of 56.8–1163.8 μg/g; manganese levels of 0.4–1.3 mg/cup) [29,30,31,32,33]. Black tea manufactured from young tender shoots of Camellia sinensis (L.) O. Kuntze is the most widely consumed non-alcoholic drink in India [29]. Hope et al. [34], concluded that tea drinking is a major source of dietary manganese and intakes commonly exceed proposed adequate intake values of 1.8–2.3 mg manganese per day and, on occasion, exceed upper limits of 10–11 mg/day.

Although neurobehavioral alterations are considered as one of the most sensitive markers of excessive exposure to manganese over long-term, there is no reported minimal manganese level that points to a chronic intoxication [28]. Hence it is difficult to estimate how much of dietary manganese intake per day and for how much duration will lead to development of neurotoxicity [28]. In addition, several studies refute the effects of dietary intake of manganese on its plasma levels [34] and on neurological functions [35]. Although arrangements for estimation of manganese concentration in drinking water used by the patient and his family were beyond our scope, none of the family members had clinical-radiological or laboratory evidence of manganism or hypermanganesemia and hence this etiology was excluded. Defect in homeostasis of bivalent ions have been associated with cognitive impairment [36,37,38,39]. However, we feel that the multi-domain (complex attention, execution, learning and memory, and social cognition) cognitive impairment in this case may be attributed to damage to cortico-striato-thalamo-cortical loop due to manganese toxicity itself [40,41].

In conclusion, this is the first reported case of acquired hypermanganesemia who presented as a combination of asymmetric chorea and cognitive dysfunction. The clinical picture mimicked that of Huntington’s disease. We highlight the potential deleterious effects of an apparently “benign” non-alcoholic beverage (i.e., black tea) on cerebral metabolism.
